# Three-dimensional reconstruction of blood vessels in the rabbit eye by X-ray phase contrast imaging

**DOI:** 10.1186/1475-925X-12-30

**Published:** 2013-04-11

**Authors:** Lu Zhang, Xiuqing Qian, Kunya Zhang, Qianqian Cui, Qiuyun Zhao, Zhicheng Liu

**Affiliations:** 1 College of Biomedical Engineering, Capital Medical University, Beijing 100069, China

**Keywords:** Three-dimensional reconstruction, Blood vessels, X-ray phase-contrast imaging, Vessel density

## Abstract

**Background:**

A clear understanding of the blood vessels in the eye is helpful in the diagnosis and treatment of ophthalmic diseases, such as glaucoma. Conventional techniques such as micro-CT imaging and histology are not sufficiently accurate to identify the vessels in the eye, because their diameter is just a few microns. The newly developed medical imaging technology, X-ray phase-contrast imaging (XPCI), is able to distinguish the structure of the vessels in the eye. In this study, XPCI was used to identify the internal structure of the blood vessels in the eye.

**Methods:**

After injection with barium sulfate via the ear border artery, an anesthetized rabbit was killed and its eye was fixed in vitro in 10% formalin solution. We acquired images using XPCI at the Shanghai Synchrotron Radiation Facility. The datasets were converted into slices by filtered back-projection (FBP). An angiographic score was obtained as a parameter to quantify the density of the blood vessels. A three-dimensional (3D) model of the blood vessels was then established using Amira 5.2 software.

**Results:**

With XPCI, blood vessels in the rabbit eye as small as 18 μm in diameter and a sixth of the long posterior ciliary artery could be clearly distinguished. In the 3D model, we obtained the level 4 branch structure of vessels in the fundus. The diameters of the arteria centralis retinae and its branches are about 200 μm, 110 μm, 95 μm, 80 μm and 40 μm. The diameters of the circulus arteriosus iridis major and its branches are about 210 μm, 70 μm and 30 μm. Analysis of vessel density using the angiographic score showed that the blood vessels had maximum density in the fundus and minimum density in the area anterior to the equator (scores 0.27 ± 0.029 and 0.16 ± 0.032, respectively). We performed quantitative angiographic analysis of the blood vessels to further investigate the density of the vessels.

**Conclusions:**

XPCI provided a feasible means to determine the structure of the blood vessels in the eye. We were able to determine the diameters and morphological characteristics of the vessels from both 2D images and the 3D model. By analyzing the images, we obtained measurements of the density distribution of the microvasculature, and this approach may provide valuable reference information prior to glaucoma filtration surgery.

## Background

Glaucoma is the first leading cause of irreversible blindness [1]. Failure of the aqueous humor (AH) outflow is the primary cause of elevated intraocular pressure (IOP) in primary open-angle glaucoma [2]. High IOP leads to retinal ganglion cell death and blindness. The current treatment for glaucoma is directed toward decreasing IOP to prevent progressive glaucomatous optic nerve damage. AH plays an important role in maintaining the balance of IOP. It is generally understood that the AH can leave the eye by two routes. The primary route is via the trabecular meshwork, which is in the angle of the anterior chamber and through Schlemm’s canal. AH leaves Schlemm’s canal either via collector channels to aqueous venous plexuses, including the scleral plexus, or Ascher’s aqueous veins, ultimately draining into the episcleral veins [3]. The other route by which AH leaves the eye is via the choroid and sclera, directly into the episcleral veins. Regardless of how it leaves the eye, the AH ultimately feeds into the vessels. An understanding of blood vessels in the eye is helpful to clinical research of glaucoma.

Conventional treatment of glaucoma is by glaucoma filtration surgery [4,5]. However, it remains unknown as to which place is best for drainage of the AH [6,7]. Whether the AH is absorbed has consequences for the safety and efficacy of aqueous shunts. By constructing a three-dimensional (3D) model of the eye vessels, our aim was to provide valuable information to glaucoma surgeons who wish to select the safest and most effective IOP-lowering procedure for each individual patient. It may also be possible to use it for analyzing hemodynamics in other eye diseases related to the eye blood supply [8].

Visualization of blood vessels is meaningful but difficult. Cardiovascular vessels have been frequently studied, but their lumen diameter is more than 1 mm [9]. The vessels in the eye are 220 μm in diameter at most. The resolution of magnetic resonance angiography (100–200 μm [10]) and that of positron emission tomography (1.5 mm [11]) are insufficient for imaging the microvascular networks. Furthermore, the vessels in the eye are complex, and a technique is required that has sufficient resolution for imaging the capillaries. Researchers prefer optical coherence tomography (OCT) to visualize the vessels in the eye [12]. However, OCT cannot image all the blood vessels in the eye at once; it can only image the retinal vessels or the blood vessels at the ocular limbus.

There are some solutions to the analysis and processing of 2D images of the retina, which are generally digital fundus photographs. The detection of vessels in the retina has improved from manual, to semiautomated, to automated [13]. Nonlinear projections [14] and multiscale line operators [15] are familiar methods to analyze retinal blood vessels. Transmission electron microscopy is able to image the deep vascular plexus of the retina [16], and blood capillaries can be visualized by lectin histofluorescence. The recently developed technique of X-ray phase-contrast imaging (XPCI) has been proposed as a new method for the imaging of the whole vasculature of eye, not only the retinal blood vessels.

Over a century ago, Roentgen discovered X-rays. Radiography is widely used for industrial and medical applications. Medical diagnostic X-rays typically have photon energies in the range from about 0.1 keV to several hundred keV, which makes X-rays an excellent probe to study structures in different media, not only biological tissues [17]. In diagnostic radiography, there are three main processes by which X-rays are transported in tissue: an X-ray photon may be absorbed, may be scattered or may traverse the tissue when it interacts with a biological sample [18]. Currently, conventional attenuation-based X-ray imaging methods dominate clinical imaging of the body. However, soft tissues are almost transparent to hard X-rays, and their visualization requires modalities such as magnetic resonance imaging. In fact, information about coherent phase changes of X-rays propagating in soft tissues may also be significant in diagnostic radiology. In the 1930s, Zernike introduced phase-contrast methods into optical microscopy [19]. A highly coherent beam (as provided by the Shanghai Synchrotron Radiation Facility, SSRF, a third-generation source of synchrotron radiation) can allow phase-contrast imaging with a very simple experimental setup [20,21].

XPCI is extensively used for the study of soft tissues. It has made possible access to information on organs and structure of insects [22]. Gao et al. studied human liver cancer and was able to visualize the guinea pig’s cochlea [23]. Lungs and lung tumors have also been widely studied [24,25]. The structure of trabecular bone [26] and the bone–cartilage interface [27] can also be distinguished by XPCI techniques. Regarding imaging of vessels, there are some relevant studies to be found in the literature. Lu et al. have obtained images of the blood vessels in rat limb muscle [21,28]. In that study, the minimum diameter of the blood vessels that could be visualized was 9 μm. Zhang et al. have achieved 3D visualization of the blood vessels [29]. As these studies demonstrate, XPCI techniques enable researchers to visualize small blood vessels. To our knowledge, XPCI has not yet been used for imaging the blood vessels in the eye or for rendering the 3D structure of the blood vessels. Therefore we propose developing a database of rabbit eye vessels using XPCI.

## Methods

### Preparation of tissues for phase-contrast imaging

The rabbits used for experiment were provided by the Experimental Animal Department of the Capital Medical University, and the animal experiments were carried out in accordance with the National Institute of Health Guide for the Care and Use of Laboratory Animals and approved by the Institutional Animal Care and Use Committee of China. A total of ten rabbits were prepared. The animal that had the eye with the most coherent and readily distinguished blood vessel morphology was selected for imaging. This rabbit had a weight of 2.8 kg. Barium sulfate was chosen as the contrast medium, and 20 g of barium sulfate was dissolved in a solution of 50 mL of glycerin and 50 mL of saline. To prevent blood clots, heparinized saline was prepared by mixing 125 MU of heparin and 500 mL of physiological saline. After injecting the rabbit with ketamine (40 mg/kg) and heparinized saline, 20 mL of contrast medium was injected via the ear border artery. After the rabbit had been killed, we excised the eye from the same side as the injection. In this experiment, the right eye was chosen. We then fixed the eye in 10% formalin solution and placed it in a refrigerator at 4°C.

### X-ray phase-contrast imaging

**Figure 1:**
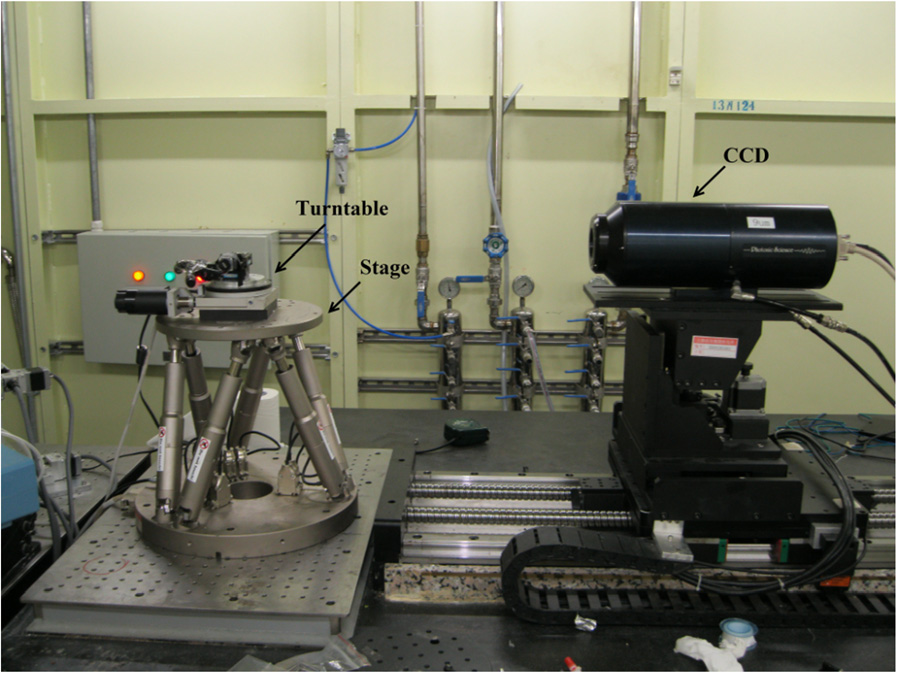
**Setup of the X-ray Imaging and biomedical application beamline unit, Shanghai Synchrotron Radiation Facility.** The stage has a six-joint control system that allows placement of the sample in the center of the field of view of the CCD. During scanning, the sample is placed on the turntable and the turntable is rotated around its cylindrical axis by 180° in steps of 1°.

The experiment was performed in the X-ray Imaging and Biomedical Application Beamline (BL13W1) at the SSRF. The setup is shown in Figure 1. The beam radiated by a wiggler, which is the 13th straight section of the SSRF, and was monochromatized by a ^111^Si double-crystal monochromator. The energy range was 8–38.1 keV. The energy and distance are important determinants of image quality. We first tested these experimental parameters, and chose 22 keV as the energy and 700 mm as the distance. A CCD camera (9 μm × 9 μm) was used as a two-dimensional detector, comprising 3,588 × 508 pixels. During scanning, the sample was rotated on a turntable around its cylindrical axis by 180° in steps of 1°. The rotation speed was 0.25° s^-1^ and the exposure time was 11 ms. Because of the beam size limit, the sample was too large to image in one scan. We divided the eye into four groups and moved the stage up and down four times in steps of 4 mm. We called the acquired images CT 1, CT 2, CT 3 and CT 4. Before and after each scan, we obtained a background image for image processing.

### Image processing

**Figure 2:**
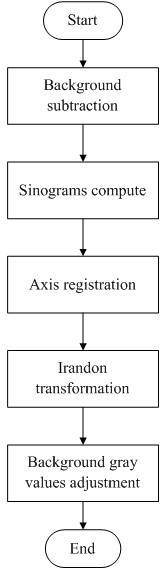
**Algorithm flowchart of the program used to convert the projected images into slices.** The sinograms were computed by FBP based on the energy, distance and number of projected images. Because of the nonuniform thickness of the tissues of the eye, the gray value of the background has not been agreed. So we adjusted the gray value of background to remove the background noise.

**Figure 3:**
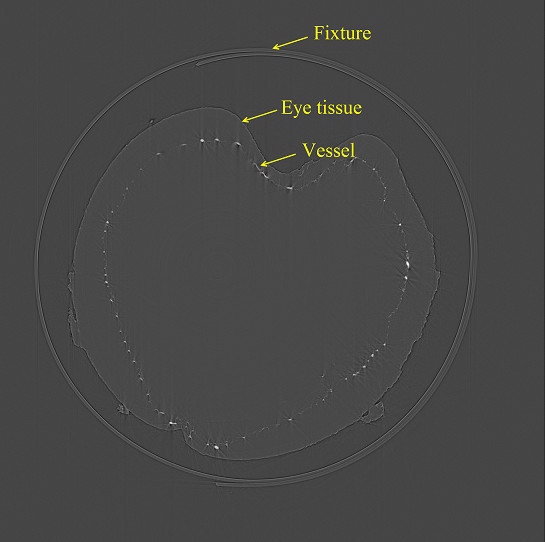
**One slice of the blood vessel database.** Projected images were converted to slices to establish a blood vessel database, and then the 3D model of the blood vessels could be obtained. The white points are cross section of the blood vessels, and the circle outside the eye is polystyrene used to fix the eye.

The background images were used for normalization during the preprocessing of the projection images. Using FBP [30], the data sets were converted at the SSRF into 1,600 slices. The algorithm flowchart is shown in Figure 2. Firstly we inputted the projected images and the background images to remove the background noise. Based on the energy, the distance and number of projected images acquired during scanning, the projected images were converted to sinograms firstly and then established the axis. By irradon transformation, we got the slices. Because of the nonuniform tissues of eye in thickness, the gray value of background was not agreed. Based on the slices obtained, we first set a gray value in the middle to remove the influence of the background on image quality. At last, the database of the slices was established. Using this algorithm, the projected images were converted to slices [31]. The number of slices was then set, and the slices were finally reconstructed. An example slice is shown in Figure 3. The 3D reconstruction was done at the SSRF by visualization software ( Amira 5.2; VSG – Visualization Sciences Group, Burlington MA, USA). Then we inputted these slices into the data pool, and selected a threshold to build the 3D model. Finally, we removed the surrounding tissues to obtain the 3D vascular model.

**Figure 4:**
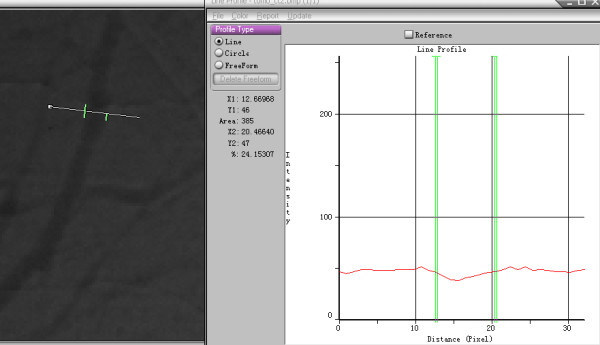
**Measurement of the blood vessels.** Left is a projected image of group CT 2. The number of pixels contained in the blood vessels was 7.8. Each pixel was 9 μm, so this blood vessel was about 70 μm in diameter. The diameter of the visualized vessels was quantified using Image-Pro Plus.

The diameter of the visualized vessels was quantified with image analysis software (Image-Pro Plus 5.0; Media Cybernetics, Rockville, MD, USA). We calculated the number of pixels included in a blood vessel. Each pixel in our study was 9 μm, which was determined by the CCD, and thus we were able to obtain the diameter of the vessel. The vessels were generally darker than the background. Figure 4 shows a projection image of CT 2, with a vertical line passing through the blood vessel. Based on the line of pixel gray values, we were able to obtain the number of pixels in the vessels. The diameter of the vessel shown in Figure 4 was about 70 μm.

### Vessel quantification

The angiographic score is widely used as a parameter to quantify the density of blood vessels in the clinical study of cancer, and it is considered a prognostic factor and therapy target in many tumors [32]. The region of interest (ROI) was selected using two different approaches. The first approach was proposed by Weidner [33]. In this approach, a few “hot spots” containing the maximal blood density are analyzed. In the second approach random representative areas are selected. The first approach is more popular because of its relative simplicity, but the second is more objective. In this study, we chose the second approach as the main analytical method. To compare the XPCI images and absorbed images, we used the first more simple approach.

**Figure 5:**
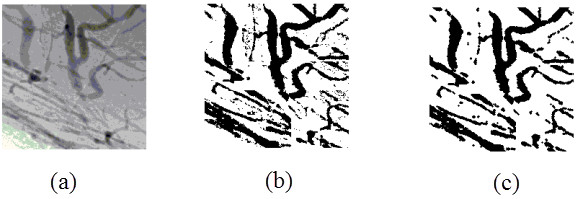
**Binary image of region of interest. (a)** The area was chosen as the region of interest. **(b)** By adjusting a suitable threshold, the image was converted to a binary image. **(c)** The result after using a morphologically open processing algorithm.

To further investigate the density of the vessels, quantitative angiographic analysis of the blood vessels was performed [34] as described previously, using a grid overlay which comprised 200 × 200 pixels divided into 25 parts, each part containing 40 × 40 pixels. First, we chose an area as the ROI and converted it into a binary image. Note that a binary image is an image composed of a 2D array of numbers that have only two values, for instance zero and one. We tried several automated methods, for example the histogram bimodal method [35], the method of Otsu [36], the K-means clustering method [37] and histogram thresholding by minimizing gray level fuzziness [38]. Because of the uneven lighting caused by the device itself and the nonuniformity of the tissues of the eye, the segmentation was not satisfied. We therefore used a semiautomated method to obtain the binary images. During this step, a global threshold was acquired by the method of Otsu to obtain an initial segmentation. We then manually adjusted the threshold according to the gray scale histogram of each part to improve the detection and segmentation of the blood vessels, just as in receiver-operator characteristic analysis [15]. The binary image of a ROI is shown in Figure 5. To remove noise further, we used a morphologically open processing algorithm to extract the vascular image, as shown in Figure 5c. A 3 × 3 structuring element object was first used to dilate the image, and then eroded it in the same way [39]. The morphologically open processing algorithm was able to remove the noise that did not belong to the vessels, rendering the blood vessels coherent.

An angiographic score was calculated as the number of pixels belonging to the vessels divided by the total number of pixels included in this area. To reduce the influence of differences in the angle, we selected three films from each angle when the turntable was at 0°, 90° and 180°. We then randomly took three different areas from each film and calculated the mean and standard deviation (SD) of the 27 scores from the same group using statistical analysis software (SPSS 17.0; IBM, Armonk, NY, USA).

### Results

**Figure 6:**
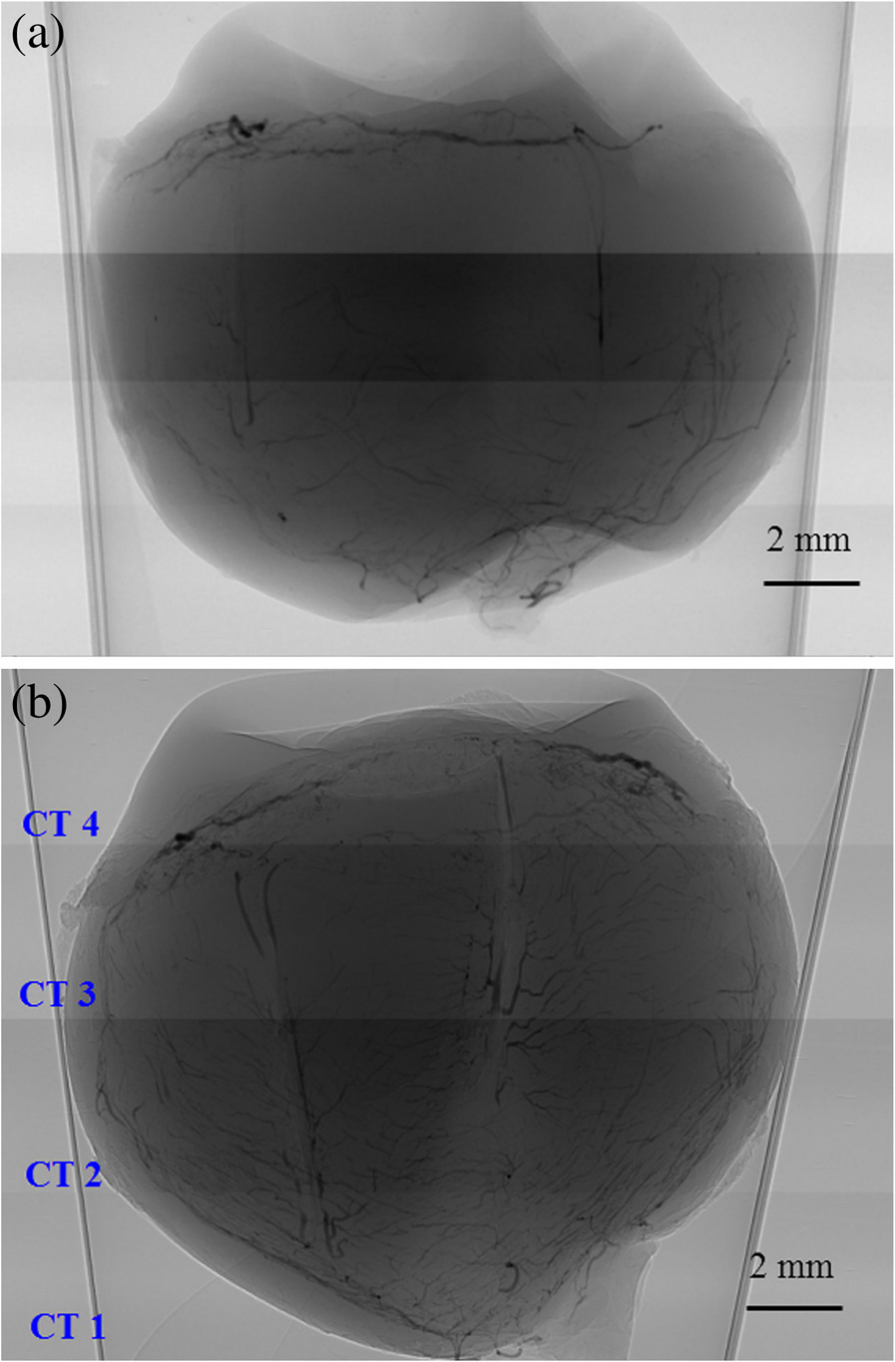
**Blood vessels in the rabbit eye are clearer in the XPCI image than in the X-ray absorption image. (a)** X-ray absorption image, in which the distance between the stage and the CCD is zero. **(b)** XPCI of the rabbit eye. The distance is 700 mm and the energy is 22 keV. Because of the limited beam size, our samples were divided the eye into four groups, CT 1, CT 2, CT 3 and CT 4.

Figure 6 shows an X-ray absorption image when the distance between the stage and the CCD is zero and the XPCI image of the rabbit eye. As can be seen, the blood vessels were more distinct and with more detail on the XPCI image than on the X-ray absorption image. The smallest vessel that could be detected on the XPCI image had a diameter of 18 μm and a sixth of the long posterior ciliary artery could be distinguished, as shown in Table 1. We randomly chose three different areas from the fundus to quantitatively analyze blood vessel density. The angiographic score of the XPCI images was higher than that of the X-ray absorption images.

**Table 1:** Comparison of data obtained from the XPCI image and X-ray absorption image

	**X-ray absorption image (n = 3)**	**X-ray phase contrast image (n = 3)**
Minimum vessel (μm)	36	18
Level of branches	5	6
Angiographic score	0.23 ± 0.021	0.30 ± 0.031^*^

Note: The angiographic score was obtained from fundus image by selecting three 200 × 200-pixel areas from each film. Values are mean ± SD. (*P < 0.05).

**Figure 7:**
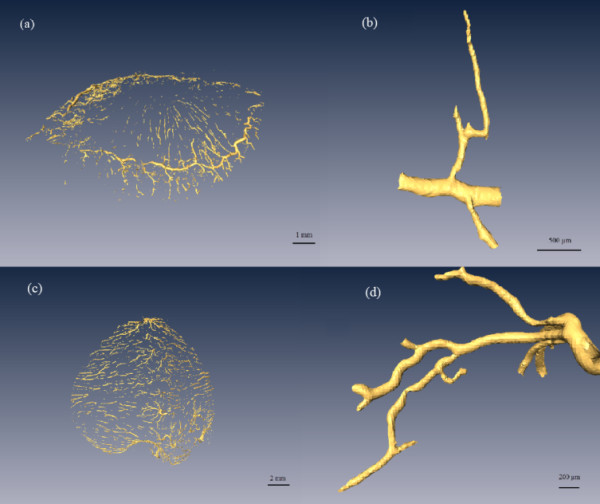
**Segments of the vascular three-dimensional model. (a)** Circulus arteriosus iridis major and its branches. **(b)** A branch of the circulus arteriosus iridis major with the minimum diameter about 30 μm. **(c)** Blood vessels of the fundus. **(d)** Branches of the fundus vessels with a minimum vessel diameter of about 40 μm.

**Figure 8:**
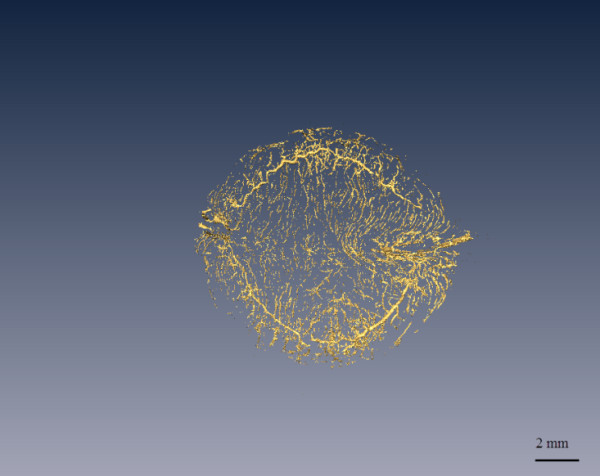
**3D reconstruction of the vasoganglion in the rabbit eye.** This figure is a vertical view from the top of the cornea to the fundus. The tissues surrounding the blood vessels have been removed. The 3D model could help observe the morphology and obtain the diameter of any vessel at any angle.

Figure 7 shows 3D reconstruction images of the vessels in the iris and fundus. Also shown are the vessel branches. The branch of the circulus arteriosus iridis major in Figure 7b is one of the radial branch vessels of the circulus arteriosus iridis major, with minimum and maximum blood vessel diameters of about 30 μm and 210 μm, respectively. Figure 7d shows the level 4 branch structure of the vessels in the fundus, where the diameters are about 200 μm, 110 μm, 95 μm, 80 μm and 40 μm. Figure 8 shows 3D reconstruction images of all vessels in the rabbit eye. We were able to visualize the spatial vascular morphology and the trend in vessels from these results, which is not possible with 2D images.

**Figure 9:**
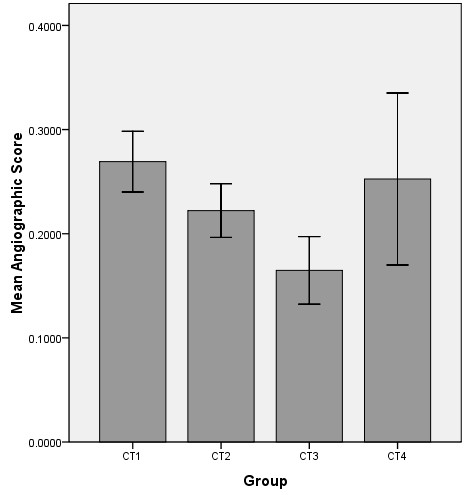
**Mean angiographic scores in the XPCI image groups.** Each group is composed of 27 ROIs and the score represents the density of the blood vessels in this region. The scores for CT 1, CT 2, CT 3 and CT 4 are 0.27 ± 0.029, 0.22 ± 0.026, 0.16 ± 0.032 and 0.25 ± 0.083 (mean ± SD), respectively.

To evaluate the density of blood vessels in the four groups, we calculated the mean and SD of the 27 scores from the same group using SPSS. The results are shown in Figure 9 and the groups are shown in Figure 6b. As shown in Figure 9, the mean angiographic score was highest in CT 1, and the widest SD range of SD was seen in CT 4, which implies that there were a greater number of blood vessels in the fundus than in the other regions and that there was a wide range of diameters of the iris vasculature. Correlation analysis revealed notable differences among the groups, and in particular, the score for CT 3 was lower than the scores for the other groups (Table 2).

**Table 2:** Correlation analysis of the angiographic scores of the groups of XPCI images

**Group**	**CT 1**	**CT 2**	**CT 3**	**CT 4**
CT 1	1	0.01**	0.00**	0.21
CT 2	0.01**	1	0.00**	0.02*
CT 3	0.00**	0.00**	1	0.00**
CT 4	0.21	0.02*	0.00**	1

Note: *P < 0.05; **P < 0.01.

### Discussion

The blood vessels inside the eyes not only show characteristic changes in response to eye diseases, but are also important in the diagnosis of diabetes, vessel occlusion and brain tumors. 3D reconstruction of eye vessels enables the analysis of the hemodynamics of blood vessels in the eye under conditions of altered IOP, which provides a meaningful reference value for the clinical study of glaucoma.

We discuss here some approaches to imaging the blood vessels in the eye. Histological sections and micro-corrosion casts are traditional methods for observing the vessels [40]. However, these methods have limited accuracy, and are usually used as standards rather than in research. Micro-CT is an alternative for imaging the blood vessels of the eye, to obtain the 3D structure and architecture of microvascular networks. 3D micro-CT has been used for visualization of the episcleral vessels by volume reconstruction [41]. However, micro-CT images are conventional attenuation-based X-ray images, and their definition is not as good as those from XPCI. Recently, OCT has been the preferred modality for 3D blood vessel reconstruction [42]. However, OCT, whether spectral domain or 3D OCT, can only image retinal vessels [43] and some groups of blood vessels in the ocular limbus [44], which is enough to diagnose diseases involving vessel occlusion or hypertension [45]. But imaging of the entire vascular system enables the distribution and trend to be viewed from all angles, and can also be used to analyze the relationship between AH flow and IOP, which may be helpful for clinical research in glaucoma.

In this study, we used XPCI to characterize in detail the blood vessels in the rabbit eye. After postprocessing, FBP translation and reconstruction, a 3D model of the blood vessel structure was obtained. Based on analysis of the pixel values, we measured the minimum blood vessel diameters in the X-ray absorption image and XPCI image and found them to be 36 μm and 18 μm, respectively. Vessel density is an index for blood vessel evaluation and is represented by the angiographic score. Diameter, morphology and density are three important indices for evaluating microvessels [46], and the discussion in this paper covers these three aspects.

Diameter is an important parameter for evaluating blood vessels. Visualization of the microvasculature is a challenge, and measurement of the diameter of the microvessels is especially difficult in the eye. A 3D model can help obtain the diameter of any vessel at any angle. If a database could be established with information about the diameter of the vessels in the eye, it could be possible to evaluate the changes in vascular caliber during the early stages of various vascular conditions. As shown in Figure 7b, the diameters of the circulus arteriosus iridis major and its branches were about 210 μm, 70 μm and 30 μm. The diameters of the arteria centralis retinae and its branches, as shown in Figure 7d, are about 200 μm, 110 μm, 95 μm, 80 μm and 40 μm. To our knowledge, there is no relevant research investigating the diameter of blood vessels in the rabbit eye, while the results are in accord with those given by Tian [46]. The vascular diameter variation follows the principle of proceeding from wide to narrow.

Morphology is another parameter used to evaluate blood vessels. We can obtain the trend and distribution of blood vessels from a 3D model. The vessels visualized using the 3D model discussed here mostly travelled in a straight line, had an even caliber, and had clear and tidy edges with smooth appearance and natural, proper proportions. No local deformities, swollen and narrow lumens, circuitousness or corkscrewing were apparent, and the distribution did not show any local or sharp reductions. Most microvessels observed in this study were dendriform, in which the blood resistance is low, which is a good morphology for exchange.

Although the mechanisms by which AH drains from the bleb after glaucoma filtration surgery have not been thoroughly elucidated [5], the structure of the microvasculature on the conjunctiva can affect the absorption of AH to a certain degree. Angiography is an important technique in current clinical medicine, and a vital approach to characterizing the microvasculature. Both in our results and in the literature [46], it can be seen that the blood vessels in the bulbar conjunctiva are dendriform. Morphology determines the physiological function of these vessels, which are responsible for local exchange, so the more blood vessels there are, the better the effect of lowering the IOP. As in glaucoma filtration surgery, anterior to the equator is usually chosen as the drainage site. However, our analysis showed that the microvasculature anterior to the equator has the fewest vessels. As shown in Figure 9, the angiographic scores for the groups CT 1, CT 2, CT 3 and CT 4 were 0.27 ± 0.029, 0.22 ± 0.026, 0.16 ± 0.032 and 0.25 ± 0.083 (mean ± SD), respectively, which means that the microvasculature in the fundus (CT 1) is the most dense and the density of blood vessels in the iris is high as well. This is expected, as usually these two places do not exhibit absorption of AH. The angiographic score for CT 2 was clearly higher than that for CT 3, so posterior to the equator of eye may be another choice for drainage. This result is consistent with the conclusions drawn from other animal experiments performed by our team [47].

A limitation of our research lies in the fact that the blood vessels in the 3D model were not completely coherent. The precipitation of barium sulfate contributes to breakpoints in the microvessels. To improve image quality, we will change the contrast agent used in a follow-up study. At the same time, we hope to develop a 3D model that can be used in a mechanical analysis, which may help the study of glaucoma and other conditions that influence the blood supply of the eyes. Another limitation of this work is that the resolution of our images was 9 μm, while the blood capillaries are smaller than that. While the theoretical spatial resolution of the system at the SSRF is about 1 μm or less, an improvement in the resolution would be at the cost of the volume of the sample that could be scanned. It would be necessary to divide the sample into further groups for scanning. Even if we are willing to make this compromise, the detector size limits the imaging field of view, and so our achievable image resolution is a balance between resolution and the size of the sample. We are inclined to favor the acquisition of the entire vascular network.

### Conclusions

In conclusion, in this study we used XPCI to obtain detailed images and a 3D model of the blood vessels in the rabbit eye. The minimum blood vessel diameter that could be distinguished in projected images was 18 μm, and the minimum and maximum diameters in the 3D model were about 210 μm and 30 μm, respectively. By analyzing the images and the 3D model, it was possible to determine some potentially useful information about glaucoma.

## Abbreviations

XPCI: X-ray phase-contrast imaging; SSRF: Shanghai Synchrotron Radiation Facility; FBP: Filtered back-projection; 3D: Three-dimensional; 2D: Second-dimensional; AH: Aqueous humor; IOP: Intraocular pressure; OCT: Optical coherence tomography; BL13W1: X-ray Imaging and biomedical application beamline; CCD: Charge-coupled device; ROI: Region of interest; SD: Standard deviation; SPSS: Statistical Product and Service Solutions; Micro-CT: Micro computed tomography; CT1: Group 1 of this paper, whose general location is the fundus; CT2: Group 2 of this paper, whose general location is the posterior to the equator of eye; CT3: Group 3 of this paper, whose general location is the anterior to the equator of eye; CT4: Group 1 of this paper, whose general location is the iris

## Competing interests

The authors declare that they have no competing interests.

## Authors’ contributions

LZ: performed the experiment and wrote the paper; QQ: contributed to discussion of the work; KZ: contributed to the experimental design; QC: finished the preparation of samples; QZ: helped LZ collect the database for the eye at the SSRF; ZL: proposed the idea and contributed suggestions throughout the work. All authors read and approved the final manuscript.
